# ^18^F-FDG PET-CT after Neoadjuvant Chemoradiotherapy in Esophageal Cancer Patients to Optimize Surgical Decision Making

**DOI:** 10.1371/journal.pone.0133690

**Published:** 2015-11-03

**Authors:** Maarten C. J. Anderegg, Elisabeth J. de Groof, Suzanne S. Gisbertz, Roel J. Bennink, Sjoerd M. Lagarde, Jean H. G. Klinkenbijl, Marcel G. W. Dijkgraaf, Jacques J. G. H. M. Bergman, Maarten C. C. M. Hulshof, Hanneke W. M. van Laarhoven, Mark I. van Berge Henegouwen

**Affiliations:** 1 Department of Surgery, Academic Medical Center, Amsterdam, the Netherlands; 2 Nuclear Medicine, Academic Medical Center, Amsterdam, the Netherlands; 3 Clinical Research Unit, Academic Medical Center, Amsterdam, the Netherlands; 4 Gastroenterlogy and Hepatology, Academic Medical Center, Amsterdam, the Netherlands; 5 Radiation Oncology, Academic Medical Center, Amsterdam, the Netherlands; 6 Medical Oncology, Academic Medical Center, Amsterdam, the Netherlands; Baylor College of Medicine, UNITED STATES

## Abstract

**Background:**

Prognosis of esophageal cancer patients can be significantly improved by neoadjuvant chemoradiotherapy (nCRT). Given the aggressive nature of esophageal tumors, it is conceivable that in a significant portion of patients treated with nCRT, dissemination already becomes manifest during the period of nCRT. The aim of this retrospective study was to determine the value and diagnostic accuracy of PET-CT after neoadjuvant chemoradiotherapy to identify patients with metastases preoperatively in order to prevent non-curative surgery.

**Methods:**

From January 2011 until February 2013 esophageal cancer patients deemed eligible for a curative approach with nCRT and surgical resection underwent a PET-CT after completion of nCRT. If abnormalities on PET-CT were suspected metastases, histological proof was acquired. A clinical decision model was designed to assess the cost-effectiveness of this diagnostic strategy.

**Results:**

156 patients underwent a PET-CT after nCRT. In 31 patients (19.9%) PET-CT showed abnormalities suspicious for dissemination, resulting in 17 cases of proven metastases (10.9%). Of the patients without proven metastases 133 patients were operated. In 6 of these 133 cases distant metastases were detected intraoperatively, corresponding to 4.5% false-negative results. The standard introduction of a post-neoadjuvant therapy PET-CT led to a reduction of overall health care costs per patient compared to a scenario without restaging with PET-CT ($34,088 vs. $36,490).

**Conclusion:**

In 10.9% of esophageal cancer patients distant metastases were detected by standard PET-CT after neoadjuvant chemoradiotherapy. To avoid non-curative resections we advocate post-neoadjuvant therapy PET-CT as a cost-effective step in the standard work-up of candidates for surgery.

## Introduction

Esophageal cancer is the eighth most common malignancy in the world, amounting to nearly half a million new cases annually [1]. Poor overall survival rates associated with esophageal cancer are mainly attributed to the aggressive nature of these tumors. Both histological subtypes, adenocarcinoma and squamous-cell carcinoma, are notorious for rapid dissemination, regionally as well as to distant sites [2].

The preferred curative strategy for patients without distant metastases consists of esophagectomy with preceding chemoradiotherapy as recent studies have shown a profound survival benefit of neoadjuvant treatment [3]. However, esophagectomy is associated with high costs, a considerable risk of severe complications and the highest mortality rate among all elective gastrointestinal surgical interventions [4]. Accurate staging at the time of diagnosis is therefore crucial to identify patients eligible for potentially curative treatment. Imperative in the preoperative phase is an accurate assessment of the M-stage since metastatic disease is an absolute contraindication for extensive surgery. Computed tomography (CT) has traditionally been used for this goal, but in the last decade fluorine-18 fluoro-2-deoxy-D-glucose (^18^F-FDG) positron emission tomography (PET) and especially the combination of these two techniques (PET-CT) have proven their superiority in metastasis detection [5–10]. Nevertheless, in a prospective multicenter study by Van Westreenen et al. the additional value of PET in initial staging of esophageal cancer was shown to be limited [11]. After conventional staging (endoscopic ultrasonography, external ultrasonography of the neck and thoracoabdominal CT) new metastases were detected by PET in only 8 of 199 included patients (4%; 95%-CI: 1.3–6.7). These results and the high costs associated with PET led to the discouragement of standardized use of PET/PET-CT at initial presentation. However, based on recent literature the value of PET-imaging after neoadjuvant treatment (restaging) is considerable [9,12,13].

During the course of nCRT (5 weeks) and recovery time (5–8 weeks) distant metastases may become manifest. Studies on these so called ‘interval metastases’ have produced convincing arguments supporting the use of post-neoadjuvant therapy PET-CT (restaging PET-CT) based on an incidence of these metastases of 8 to 17% [14–16]. Despite these results the use of diagnostic modalities to detect interval metastases is not considered standard of care. The present study evaluates the clinical value, diagnostic accuracy and cost-effectiveness of PET-CT imaging after neoadjuvant chemoradiation for esophageal cancer in order to prevent non-curative surgery on patients with distant metastases.

## Methods

### Patient population

Between January 2011 and February 2013 all consecutive esophageal cancer patients deemed eligible for a curative approach with nCRT and surgical resection were included in the present retrospective study. Electronic charts were available for all these patients and contained information regarding diagnostic work-up, treatment and treatment outcomes in terms of toxicity, complications and long-term follow-up. Individual treatment strategies were defined during a multidisciplinary treatment meeting in which gastroenterologists, medical oncologists, radiation oncologists, radiologists, nuclear medicine physicians, pathologists and gastrointestinal surgeons participated. Initial staging consisted of endoscopy with biopsy, endoscopic ultrasonography, external ultrasonography of the neck and a thoracoabdominal CT scan. A PET-CT scan was not part of the initial staging, but was performed in a few cases by referring physicians. Neoadjuvant chemoradiotherapy followed by esophagectomy was indicated in patients deemed fit for surgery with histologically proven, locally advanced, resectable malignancy without distant metastases (cT1N+M0 or cT2-3N0-3M0). Patients who were unable to complete neoadjuvant treatment due to toxicity but were fit for surgery were included in the eventual analysis. Patients were not asked to provide informed consent for this specific study because the used data was primarily recorded as part of standard care. On arrival at our outpatients clinic, patients were informed that data collected as part of standard care could be used for scientific purposes. Patients did have the opportunity to refuse permission to use their medical information for this goal. The local ethics committee of the Academic Medical Centre Amsterdam approved this approach.

### Treatment

Two neoadjuvant chemoradiotherapy regimens were employed in the present study. All patients received 23 fractions of 1.8 Gy (41.4 Gy) external-beam radiotherapy combined with concurrent weekly administered carboplatin (AUC2) and paclitaxel (50 mg/m^2^) in accordance with the recently published CROSS trial [3]. Additionally, as part of a phase II clinical trial in our centre, a proportion of patients received panitumumab (human monoclonal antibody to the epidermal growth factor receptor) at a dose of 6 mg/kg in addition to standard neoadjuvant chemoradiation [17]. Esophagectomy was performed within 5 to 8 weeks after completion of nCRT using either a (convential or minimally invasive) transthoracic or transhiatal approach as described in previous reports [18,19]. Postoperative follow-up occurred in accordance with the Dutch guideline and consisted of frequent clinical evaluations by a surgeon and imaging in case of suspected recurrent disease.

### PET-CT imaging

Restaging PET-CT was scheduled 3 weeks after completion of neoadjuvant chemoradiotherapy. The PET-CT was performed using a Philips Gemini TF-16 PET/CT scanner (Philips Medical Systems, Eindhoven, The Netherlands) with spatial resolution near the field of view center of 4.8 mm in transverse and axial directions. A CT scan in the supine position was acquired from the base of the skull to mid-thighs. The 12-channel helical CT scanning parameters were: 120 kVp, 50 mA/slice, rotation time 0.75 seconds, and slice thickness/interval 3.0 mm. Both oral and intravenous (porto-venous phase) contrast was used. At 60 minutes after intravenous injection of 180–240 MBq of 18F-FDG, emission scans were acquired from the base of the skull to mid-thighs over 10 bed positions at 2 minutes per position. Image reconstruction employed a list-mode version of a maximum likelihood expectation maximization algorithm with a time-of-flight kernel applied in both the forward and back-projection operations. Quantitative analysis was performed using standardized uptake values (SUVs) and calculated as the maximum value 1 hour after injection. CT data were used for attenuation correction. Images were viewed using Hermes Hybrid viewer software (Hermes Medical Solutions, Stockholm, Sweden). Foci of abnormal FDG uptake greater than that of background activity were considered suspected metastases. Cases in which restaging PET-CT led to this suspicion were re-evaluated in a multidisciplinary meeting to consider available options to obtain histological or cytological proof. Patients with histologically confirmed metastatic disease were excluded from surgical resection and were referred for palliative treatment.

### Re-evaluation of initial imaging

Baseline thoracoabdominal CT imaging of patients with proven metastatic disease was systematically reviewed for metastatic disease by an independent and experienced gastrointestinal radiologist to determine to what extent lesions were already manifest before start of the neoadjuvant therapy. This revision took place in two phases: at first without knowledge of the restaging PET-CT results and subsequently after disclosure of the location(s) of metastases. Similarly, an independent and experienced gastrointestinal nuclear medicine physician revised restaging PET-CT images of patients with metastatic disease detected intraoperatively in order to determine to what extent lesions were manifest before surgery. This revision also took place with and without prior knowledge of the location(s) of metastatic disease.

### Statistical methods

In order to determine the accuracy of restaging PET-CT in identifying metastatic disease, sensitivity and specificity were calculated. These calculations were patient- instead of lesion-based, which means that PET-CT was considered true positive if findings led to justified cancellation of surgery because of the presence of metastatic disease. PET-CT was scored true negative if the absence of metastatic disease was confirmed during surgical exploration. If PET-CT led to the suspicion of distant metastases while in fact no metastatic disease was present, imaging was considered false positive. Finally, PET-CT was deemed false negative if metastases were detected during surgical exploration.

For the purpose of cost-effectiveness analysis, the costs of diagnostic strategies and treatment were examined from a provider perspective (costs borne by the hospital), focusing on the personnel, material and overhead costs. The mean costs of additional diagnostics after restaging PET-CT were included in the analysis. Based on these costs and the observations in our cohort a clinical decision model was built with Data TreeAge Software [20]. In this decision model two clinical scenarios (no restaging imaging and restaging imaging by PET-CT) were compared based on the calculation of extra costs per additional correctly identified case of surgical eligibility and on the calculation of the average costs per patient overall. Correctly identified cases were defined as: surgery in patients without metastatic disease and cancellation of surgery in patients with metastatic disease. A threshold analysis was performed to identify the maximum costs for additional diagnostics, in which case both scenarios generate equal costs per correctly identified case. The time horizon of the analysis equaled the length of the current disease episode until the decision regarding surgery was taken and surgery was performed, if indicated.

Patients who were deemed eligible for surgery based on restaging PET-CT but did not undergo an operation were excluded from both the calculation of diagnostic accuracy and the cost-effectiveness analysis.

## Results

### Study population

**Fig 1:**
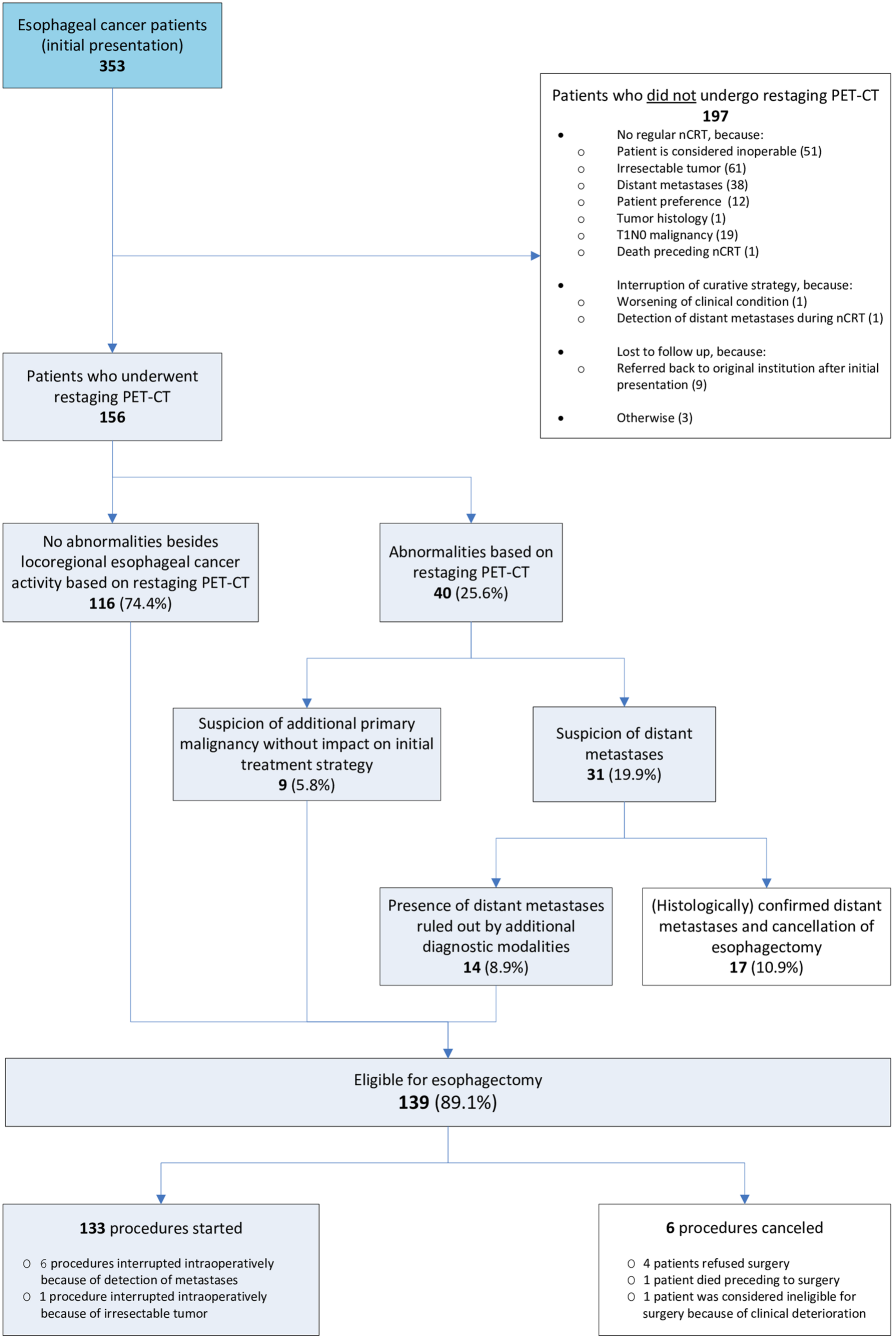
Flowchart of clinical course of patients who underwent restaging PET-CT.

From January 2011 to February 2013, 353 newly diagnosed esophageal cancer patients were analyzed at the Academic Medical Center in Amsterdam. The clinical course of these patients is represented in Fig 1. Based on the results of initial staging and assessments of physical condition a curative treatment strategy consisting of nCRT and esophagectomy was initiated in 158 of the 353 patients (44.8%). 195 patients were considered ineligible for this approach for a variety of reasons (Fig 1). Curative treatment was ceased during the neoadjuvant course in two patients (1.3%) because of deteriorating clinical condition or detection of distant metastases. The remaining 156 patients (98.7%) underwent a restaging PET-CT with a median interval from the initial CT scan of 69 days and a median interval from the last day of nCRT of 18 days. Characteristics of the 156 patients who underwent restaging PET-CT are described in Table 1.

**Table 1:** Characteristics of patients who underwent restaging PET-CT in the work-up for potentially curative esophagectomy.

Patients’ characteristics		*N* = 156
Age, median (range)		65 (34–83)
Sex, male (%)		119 (76.3%)
Tumor location		
	Middle thoracic (%)	12 (7.7%)
	Lower thoracic (%)	101 (64.7%)
	Esophagogastric junction / cardia (%)	43 (27.6%)
Tumor histology		
	Adenocarcinoma (%)	126 (80.8%
	Squamous cell carcinoma (%)	29 (18.6%)
	Other (%)	1 (0.6%)
AJCC Stage [31]		
	IB (%)	5 (3.2%)
	IIA (%)	7 (4.5%)
	IIB (%)	38 (24.4%)
	IIIA (%)	55 (35.3%)
	IIIB (%)	36 (23.1%)
	IIIC (%)	11 (7.1%)
	Unknown (%)	4 (2.6%)
Neoadjuvant therapy regimen		
	Radiotherapy (41.4Gy), carboplatin, paclitaxel (%)^a^	139 (89.1%)
	Radiotherapy (41.4Gy), carboplatin, paclitaxel, panitumumab(%)^b^	17 (10.9%)

AJCC, American Joint Committee on Cance

^a^ including 6 cases of incomplete neoadjuvant treatment due to toxicity

^b^ treatment as part of a phase II clinical trial

### Outcome of restaging PET-CT

**Fig 2:**
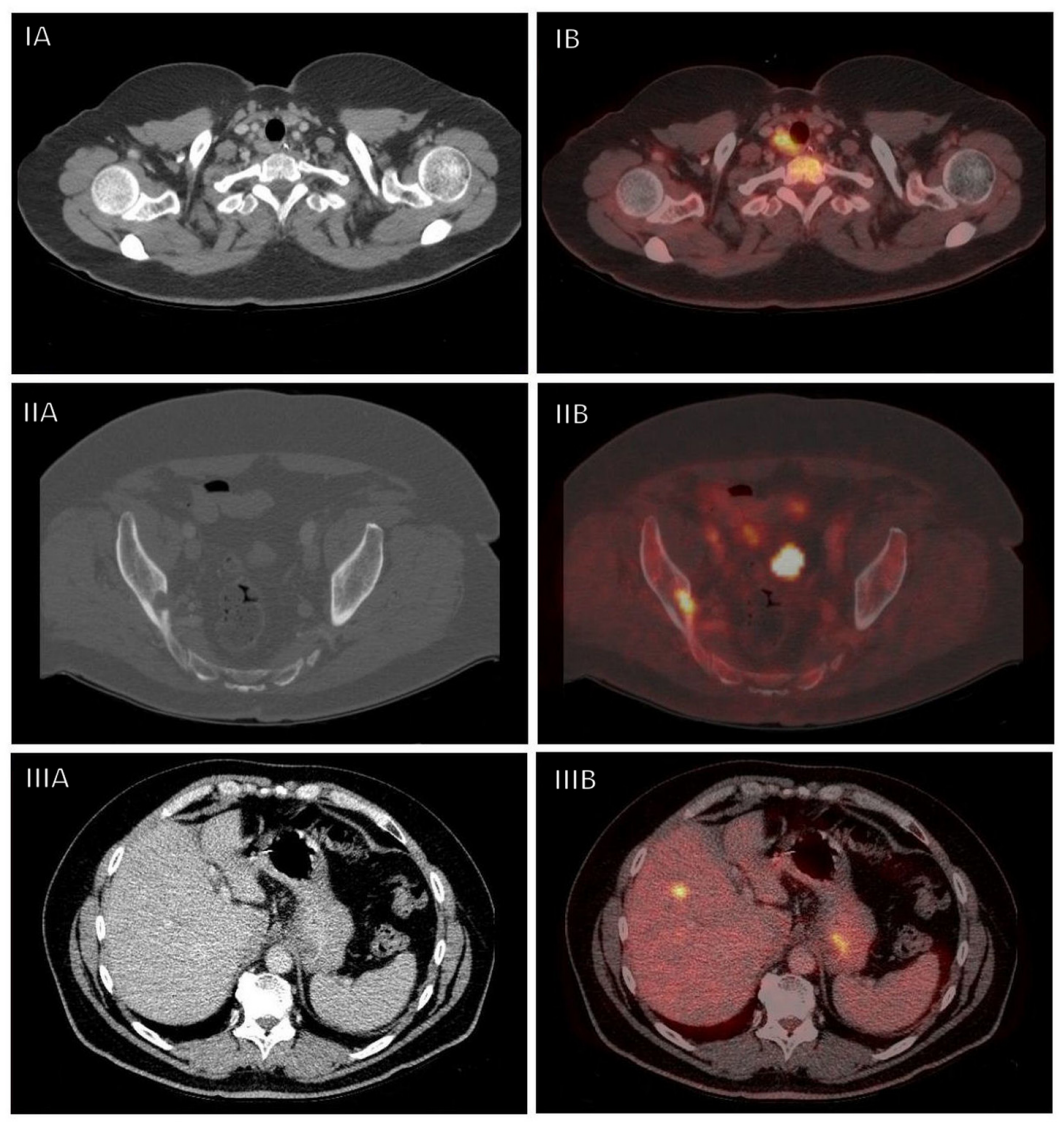
Examples of metastatic disease detected with restaging PET-CT. Examples of interval metastases on restaging PET-CT located in a supraclavicular lymph node (I), the right iliac body (II) and the liver (III). Panel A and B represent axial CT images before and after fusion with PET, respectively.

In 31 out of 156 patients (19.9%), restaging PET-CT identified possible metastases of esophageal carcinoma. In 2 of these cases the presence of metastases was evident to such a degree that no further pathological confirmation was sought (Table 2, patient 1 and 2, diffuse sclerotic bone lesions). In 15 patients metastases were confirmed by additional imaging and biopsy (Table 2, patients 3–17), leading to a total number of 17 cases of confirmed metastases (10.9%). Even in retrospect and with knowledge of the location of the metastases, only two of these lesions (patients 9 and 13) appeared to be detectable during reevaluation of the baseline thoracoabdominal CT. The median interval between initial staging and detection of these metastases was 71 days. Examples of detected interval metastases are shown in the axial CT- and fuses PET-CT images of Fig 2.

**Table 2:** Demographic and clinical details of patients with metastases found on restaging PET-CT.

Patient no.	Age (y)	Sex	Tumor location	Tumor histology	AJCC stage [31] at baseline	Neoadjuvant regimen	Location of PET-positive lesion	Additional diagnostics (pathological findings)
1	68	F	LT	Adenocarcinoma	3A	41.4 Gy, carboplatin, paclitaxel	Bone (multiple lesions)	n/a
2	54	M	LT	Adenocarcinoma	3A	41.4 Gy, carboplatin, paclitaxel	Bone & liver (multiple lesions)	n/a
3	55	M	LT	Adenocarcinoma	3A	41.4 Gy, carboplatin, paclitaxel	Scapula	CT-guided biopsy (histological confirmation)
4	69	F	LT	Squamous cell carcinoma	2B	41.4 Gy, carboplatin, paclitaxel	Supraclavicular lymph node	US-guided puncture (cytological confirmation)
5	54	M	LT	Squamous cell carcinoma	3C	41.4 Gy, carboplatin, paclitaxel	Kidney	US-guided puncture (cytological confirmation)
6	68	M	EGJ	Adenocarcinoma	Inconclusive	41.4 Gy, carboplatin, paclitaxel	Para-aortic lymph nodes	Diagnostic laparotomy (histological confirmation)
7	57	F	LT	Adenocarcinoma	3C	41.4 Gy, carboplatin, paclitaxel	Liver	US-guided puncture (cytological confirmation)
8	77	M	LT	Adenocarcinoma	2B	41.4 Gy, carboplatin, paclitaxel	Retroclavicular lymph node	US-guided puncture (cytological confirmation)
9	64	M	LT	Squamous cell carcinoma	2A	41.4 Gy, carboplatin, paclitaxel	Adrenal gland	US-guided puncture (cytological confirmation)
10	73	M	EGJ	Adenocarcinoma	3A	41.4 Gy, carboplatin, paclitaxel	Lymph node near renal vein	CT-guided puncture (cytological confirmation)
11	62	F	LT	Squamous cell carcinoma	3A	41.4 Gy, carboplatin, paclitaxel	Liver	US-guided biopsy (histological confirmation)
12	34	F	EGJ	Adenocarcinoma	3C	41.4 Gy, carboplatin, paclitaxel	Supraclavicular lymph node	US-guided puncture (cytological confirmation)
13	46	M	LT	Adenocarcinoma	3B	41.4 Gy, carboplatin, paclitaxel, panitumumab	Iliac crest	CT-guided biopsy (histological confirmation)
14	65	M	EGJ	Adenocarcinoma	3A	41.4 Gy, carboplatin, paclitaxel	Iliac body	CT-guided biopsy (histological confirmation)
15	65	M	LT	Adenocarcinoma	3B	41.4 Gy, carboplatin, paclitaxel, panitumumab	Para-aortic lymph node	CT-guided puncture (cytological confirmation)
16	51	M	LT	Adenocarcinoma	3C	41.4 Gy, carboplatin, paclitaxel	Liver	US-guided puncture (cytological confirmation)
17	69	M	LT	Adenocarcinoma	2B	41.4 Gy, carboplatin, paclitaxel	Iliac body & sacrum	Renewed PET-CT (biopsy not achievable)

Y, years; M, male; F, female; LT, lower thoracic; EGJ, esophagogastric junction; AJCC, American Joint Committee on Cancer; US, ultrasonograpy; PET, positron emission tomography; CT, computed tomography; n/a, not applicable

In the remaining 14 cases (8.9%) PET-CT based suspicion of metastatic disease was rescinded after additional imaging and biopsy. During surgical exploration and postoperative follow-up these lesions remained unsuspected. In 9 patients (5.8%) restaging PET-CT revealed potentially malignant lesions that could not be connected to esophageal cancer based on their anatomical location. These lesions were situated in the large intestine (5 out of 9), hypopharynx (3 out of 9) and the parotid gland (1 out of 9). All 9 patients were further analyzed, leading to one confirmed case of a second primary malignancy: a spindle cell carcinoma of the hypopharynx.

After deducting 17 events of detected (interval) metastases, 139 out of 156 patients (89.1%) were considered eligible for esophagectomy after restaging PET-CT. 4 of these patients receded from their initial decision and refused an operative intervention, 1 patient died of pneumonia before surgery and in 1 patient surgical exploration was canceled because of clinical deterioration with a benign origin shortly before the procedure. In 6 of the remaining 133 patients metastatic disease was detected and histologically confirmed intraoperatively (Table 3), leading to 4.5% false-negative results. The mean time between restaging PET-CT and surgical exploration for these 6 patients was 52.8 days versus 42.9 days for the remaining patients. In 3 of the 6 patients with a false-negative PET-CT (patients 1, 2 and 6) PET-CT had already resulted in suspicion of metastatic disease. However, this suspicion was based on other locations than the actual metastatic sites and was therefore rescinded after biopsy. One pulmonary metastasis (patient 6) was (in retrospect) visible during reevaluation of the PET-CT. However, because of the small size of the particular lesion no FDG-uptake was observed and cytological puncture would have been impossible.

**Table 3:** Demographic and clinical details of patients with metastatic disease detected intraoperatively in spite of ‘M0’ restaging on PET-CT.

Patient no	Age (y)	Sex	Tumor location	Tumor histology	AJCC stage [31] at baseline	Neoadjuvant regimen	Restaging PET-CT findings	Location & size of metastases	Esophagectomy cancelled due to intraoperative findings
1	66	M	LT	Adenocarcinoma	3A	41.4 Gy, carboplatin, paclitaxel	Suspicion of metastasis 2^nd^ rib (ruled out by biopsy)	Liver (10mm)	No
2	52	M	LT	Adenocarcinoma	2B	41.4 Gy, carboplatin, paclitaxel	Suspicion of metastasis cervical lymph node (ruled out by puncture)	Lung (6–7 mm)	Yes
3	77	M	LT	Adenocarcinoma	1B	41.4 Gy, carboplatin, paclitaxel	M0	Liver (4-10mm)	Yes
4	72	M	EGJ	Adenocarcinoma	3A	41.4 Gy, carboplatin, paclitaxel	M0	Multipele pleural lesions < 1 mm	Yes
5	64	M	EGJ	Adenocarcinoma	3B	41.4 Gy, carboplatin, paclitaxel	M0	Liver (5–10 mm)	Yes
6	75	M	LT	Adenocarcinoma	3B	41.4 Gy, carboplatin, paclitaxel	Suspicion of metastasis left femur (ruled out by biopsy)	Lung (3–4 mm)	Yes

Y, years; M, male; LT, lower thoracic; EGJ, esophagogastric junction; AJCC, American Joint Committee on Cancer; PET, positron emission tomography; CT, computed tomography

Based on these observations, sensitivity and specificity for restaging PET-CT with respect to metastatic disease are 73.9% and 91.3% respectively.

### Cost-effectiveness analysis

**Fig 3:**
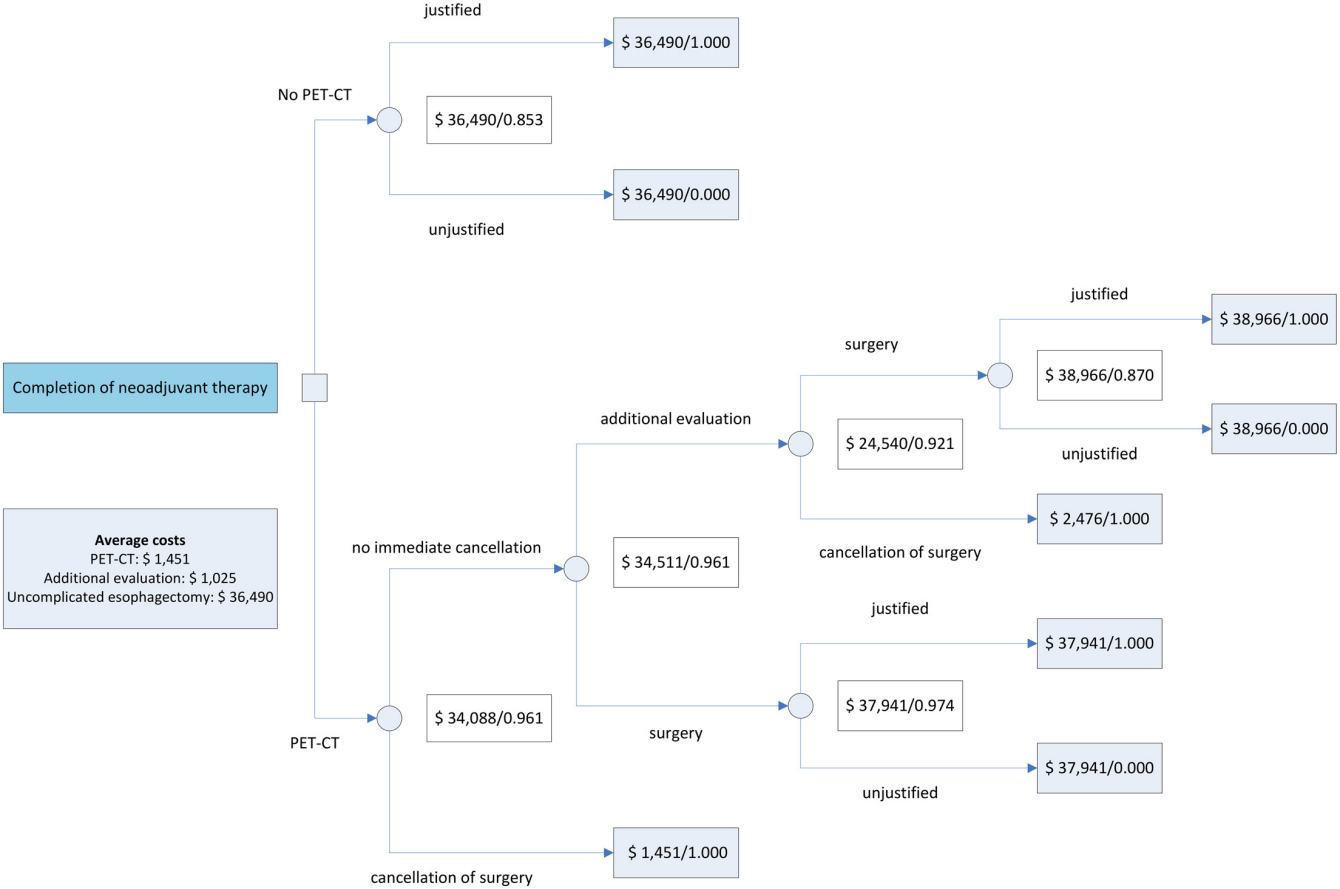
Decision model reflecting a non-restaging scenario and a scenario in which restaging with PET-CT is performed.

In a direct comparison of scenarios restaging by PET-CT led to clinically justified decisions in 96.1% of cases versus 85.3% when no restaging diagnostics were used (Fig 3). The average costs per patient decreased by $2,402 when restaging PET-CT was used, which corresponded to a saving of $7,327 per correctly identified case of surgical eligibility. Based on these proportions the costs of additional evaluation after PET-CT could increase up to $28,854 before restaging by PET-CT became less efficient than a strategy in which no restaging diagnostics were used.

## Discussion

The results of the present study indicate that (interval) metastases are detectable in more than 10% of esophageal cancer patients who receive neoadjuvant chemoradiotherapy. With a sensitivity and specificity of 73.9% and 91.3% respectively, PET-CT is an accurate tool to identify these cases. In these patients an esophagectomy can be avoided. Patients with (interval) metastases do not benefit from surgical resection in terms of survival, but merely lose quality of life because of surgery related morbidity and prolonged recovery time even if uneventful. Furthermore, surgical complications could limit palliative treatment options and precipitate cancer related death [21]. The reported sensitivity and specificity are consistent with the results of recent meta-analyses on the performance of PET-CT in detecting metastases of esophageal carcinoma at initial staging [12,13,22].

Despite the high costs of PET-CT imaging and possible additional diagnostic procedures, restaging eventually leads to financial savings because expensive non-curative resections followed by a substantial hospital admission are prevented. To our knowledge, this is the first report on cost-effectiveness of restaging imaging (by PET-CT) in an esophageal cancer setting. Malik et al. recently described the incidence and costs of PET-detected synchronous primary tumors during the initial staging of esophageal cancer [23]. In their cohort of 591 patients an additional malignancy was suspected in 9.3% of cases leading to a modest increase of extra diagnostic costs ($44.80 per patient).

Currently an unequivocal restaging protocol is still absent, even though previous reports on this topic have shown incidence rates of interval metastases between 8%-17% [14,15,24–26]. Presently, two reports have addressed the use of PET-CT in detection of interval metastases as a primary topic. In a recent study by Blom et al. 4 cases of interval metastases were detected in a consecutive series of 50 neoadjuvantly treated patients (8%) [14]. Restaging PET-CT took place 6 weeks after completion of neoadjuvant therapy that consisted of 5FU, cisplatinum and 50.4 Gy radiotherapy. A false-positive rate of 2% was reported in this cohort and in 1 out of 46 patients (2.2%) metastatic disease was observed intraoperatively [14].

In another study on restaging PET-CT the records of 85 patients treated either with induction chemotherapy followed by concurrent chemoradiotherapy or with concurrent chemoradiotherapy only were retrospectively reviewed [15]. A total number of 7 cases (8%) of interval metastases was described without disclosure of false-negative or false-positive rates. Apart from smaller patient cohorts, both studies were conducted in an era before the results of the CROSS trial were known and therefore different neoadjuvant regimens were administered [3]. The tested regimen of carboplatin, paclitaxel en concurrent radiotherapy is becoming standard of care in an increasing part of the world.

In the present cohort PET-CT based suspicion of metastatic disease was rejected after additional evaluation in 8.9% of patients. False-positive results involve considerable expenses and could delay treatment. The mentioned risk of false-positive results could be an overestimation because of false-negative biopsies.

A lean cost-effectiveness analysis was performed. Considering the clear dominance of the strategy of restaging with PET-CT over a strategy without PET-CT—cheaper and more effective at the point estimates—an extensive probabilistic sensitivity analysis for the incremental cost-effectiveness ratio was discarded. The performed threshold analysis for the costs of additional diagnostics confirmed that applying restaging with PET-CT left ample room for further optimizing this diagnostic trajectory of screening patients for surgery.

A considerable subject of discussion within this study is the timing of restaging PET-CT. In accordance with the CROSS trial protocol patients underwent surgery as soon as possible after completion of nCRT, preferably within 6 to 8 weeks [3]. In order to meet this time limit, even if additional diagnostic procedures were indicated, restaging PET-CT was scheduled 3 weeks after completion of nCRT. However, it is known that during the first weeks following nCRT a falsely positive signal is frequently detected by PET due to local and systemic inflammation from chemoradiotherapy and tumor necrosis. A longer interval between nCRT and PET-CT would therefore improve the diagnostic accuracy of PET-CT. Studies by Ruol et al and Kim et al have proven that a longer waiting period between nCRT and surgery is safe and does not affect oncologic outcome [27,28]. Based on these findings postponement of surgery is increasingly common which could allow for a more optimal timing of restaging PET-CT, preferably after the 12^th^ week post-nCRT.

The absence of PET-CT at initial staging represents another limitation of this study. In order to identify true interval metastases the same imaging technique should be used before and after neoadjuvant treatment. Additionally, the use of PET-CT both at baseline and after nCRT would enable response evaluation. Recent reports on this topic have shown that suchlike response assessments may correlate with clinical outcome and therefore can be used to further optimize surgical decision making [16,29,30]. Financial reasons have precluded the possibility of two PET-CT’s per patient and have led to the proposed work-up, which is supported by the mentioned findings of van Westreenen et al [11]. Additionally, it may be hypothesized that a restaging CT scan could have been sufficient to detect metastatic disease. This would have further increased the cost-effectiveness of the restaging procedure. However, based on available literature PET is currently recommended to improve the accuracy of M staging [7]. A meta-analysis from 2008 showed that PET has a 71% sensitivity and a 93% specificity in the detection of distant metastases in comparison to 52% and 91% for CT, respectively [12]. In summary, the outcomes of this study indicate the clinical relevance and cost-effectiveness of accurate restaging after neoadjuvant therapy for resectable esophageal carcinoma. Given the high impact, both clinically and financially, we plead for the use of PET-CT as the most sensitive imaging technique to guide preoperative decision-making and to avoid non-curative esophagectomies.
